# Relationship between End-Tidal CO_2_ (ETCO_2_) and Lactate and their Role in Predicting Hospital Mortality in Critically Ill Trauma Patients; A Cohort Study

**DOI:** 10.30476/BEAT.2020.46447

**Published:** 2020-04

**Authors:** Elham Safari, Mehdi Torabi

**Affiliations:** 1 *Department of Emergency Medicine, Kerman University of Medical Sciences, Kerman, Iran*

**Keywords:** Capnography, Hospital mortality, Lactate, Multiple trauma

## Abstract

**Objective:**

To investigate the relationship between end-tidal CO_2_ (ETCO_2_) and serum lactate and their predictive role in hospital mortality of intubated multiple trauma patients.

**Methods:**

In a cohort study, intubated multiple trauma patients who referred to the emergency department for two years were enrolled. After orotracheal intubation using Rapid Sequence Intubation (RSI) method, ETCO2 was immediately measured by capnography. Blood samples for serum lactate measurements were sent to the laboratory, immediately after intubation. Data collection was done using the questionnaire, and the patients were followed using their medical records.

**Results:**

Totally, 250 patients were included with hospital mortality of 14.8% (n=37). Using Pearson correlation, an inverse relationship was noticed between serum lactate and ETCO2, immediately (p<0.0001, r=-0.65). In adjusted multivariate analysis, three variables including heart rate (HR), serum lactate and ETCO2 showed a significant relationship with hospital mortality, respectively (p=0.007, p=0.009, p=0.023, respectively). Receiver operating characteristic curve illustrated an area under the curve (AUC) of 0.93, 0.96, and 0.97 for HR, lactate, and ETCO2, respectively.

**Conclusion:**

ETCO_2_ post-intubation and serum lactate may be considered as prognostic factors for intubated multiple trauma patients referring to the emergency department, which can give the clinician an important clue in early prediction of the hospital mortality.

## Introduction

Multiple trauma is considered as a universal health concern and it can be a threat to the patient survival rate. The term refers to the life-threatening injuries in single or multiple organs with Injury Severity Score (ISS) greater than 16, which is considered as one of the main causes of hospital mortality [1-3]. Eighty percent of hospital mortalities for multiple trauma patients occur in the first 4 hours of arrival to the emergency department. Therefore, early diagnosis and appropriate management in the emergency departments are very important [4]. Metabolic and physiologic factors may have a good predictive value in these circumstances. Multiple scoring systems, along with traditional vital signs, are helpful in determining the patient’s outcomes [1-4]. One of these measurements is the non-invasive end-tidal CO_2 _(ETCO_2_) measurement used along with capnography. ETCO_2_ is the maximum concentration of carbon dioxide at the end of exhalation, which is in direct relationship with the cardiac output [5]. 

The decrease occurred in cardiac output and inducing hemodynamic instability leads to the decline in the pulmonary blood circulation and ETCO_2_. An increase in tissue perfusion increases the ETCO_2_. This is a valuable measure to assess patients’ metabolic condition. ETCO_2_ is inversely related to metabolic acidosis, although there is only a moderate correlation between PCO_2_ and ETCO_2_ levels [6, 7]. Therefore, the use of ETCO_2_ may have the prognostic role in determining the hospital mortality in the emergency department, although this role is unclear in patients with multiple trauma [8-11]. In patients with acute conditions such as trauma, with multifactorial causes and different mechanisms, serum lactate levels increase. Considering that the normal vital signs cannot rule out hypoperfusion and shock, elevating serum lactate levels can suggest tissue hypoperfusion [12]. Serum lactate is a low cost, fast, and simple test that can predict the severity of damage and mortality in multiple trauma patients [13]. While there is an inverse relationship between ETCO_2_ and serum lactate in patients with suspected sepsis [14,15], this association is unclear in intubated multiple trauma patients. The aim of this study was to investigate the relationship between ETCO_2_ and serum lactate and their predictive role in hospital mortality of intubated multiple trauma patients. 

## Materials and Methods

*Study population*

This prospective cohort study was conducted on intubated multiple trauma patients who were admitted in the emergency department of Bahonar Academic Hospital, Level II Trauma Center, affiliated to Kerman University of Medical Sciences, Kerman, southeastern Iran. All intubated multiple trauma patients with ISS≥16 and older than 18 years old from March 1^st^, 2016 to March 1^st^, 2018 were enrolled. Upon arrival at the emergency department, all patients were triaged by a nurse. Non-intubated patients with an ISS<16, who aged less than 18 years old, patients with delayed arrival after an hour, patients who had died before entering the hospital, those patients who had been intubated prior to their admission to the hospital were excluded from the study. Furthermore; those patients who were pregnant, patients with a history of the chronic pulmonary disease, patients with drug and alcohol poisoning, seizure, organs failure, sepsis and also patients who were intubated through RSI method using different drugs were also excluded from the study. This study was based on the Helsinki Statement of 1975, which was revised in 1989 in Hong Kong and it was approved by the Ethics Committee of Kerman University of Medical Sciences, Kerman, Iran (IR.KMU.REC.1396.1530). Written, informed consent was taken from all patients before the study and all information remained confidential with the researcher. 

*Study protocol*

Patients on the 1^st^ and 2^nd^ triage levels were transferred immediately to the resuscitation room and were visited by a post-graduate 3^rd^ year (senior resident). Patients would be evaluated according to the Advanced Trauma Life Support (ATLS) guideline, and if necessary, an appropriate airway was provided through orotracheal intubation using the *Rapid Sequence Intubation* (RSI) method based on an identical protocol for all patients to avoid random measurement error. Immediately after intubation, ETCO2 was checked by sidestream capnography (IRMA CO2 Probe). Immediately after intubation, blood samples were sent to the laboratory for measurement oof serum lactate in tubes containing EDTA and lactate oxidase enzyme method was applied for biochemical analysis using the mindray device. Other variables such as age, sex, vital signs, Arterial Blood Gas (ABG), Injury Severity Score (ISS) and Revised Trauma Score (RTS) were recorded by a senior resident in a pre-prepared questionnaire for patients. The hospital mortality rate as an outcome for these patients was investigated by using medical records.

*Statistical analysis*

For quantitative variables, mean ± SD were used and for qualitative variables, the percentage of frequency was applied. To review the relationship between serum lactate and ETCO_2_, Pearson correlation was used. To express the severity of the association, the Odds Ratio (OR) and Confidence Interval (CI) 95% were employed. First, univariate analysis was applied to find statistically significant associations. Then for variables with *p*<0.25, multivariate analysis with a logistic regression model to find a significant correlation was used (*p*<0.05) [16]. Finally, for variables that had a significant relationship with the outcome, Receiver Operating Characteristic (ROC) curve was designed. Statistical analysis was done by SPSS software (version 20, Chicago, IL, USA).

## Results

**Fig. 1:**
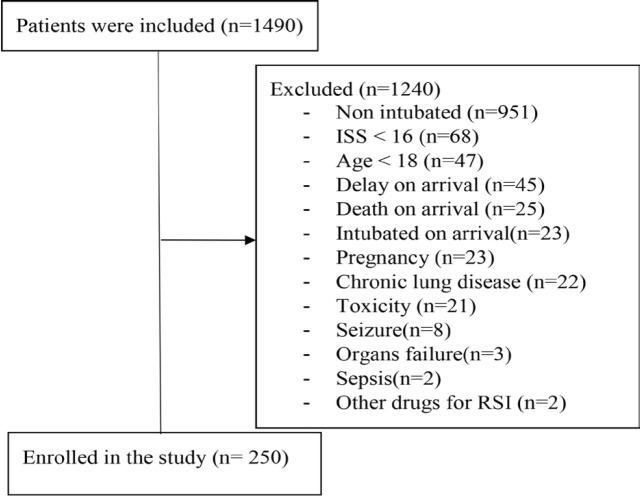
Flow chart showing enrollment of patients

**Fig. 2:**
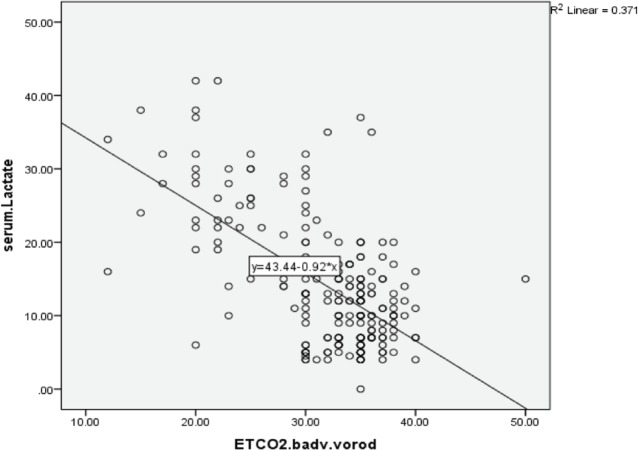
Correlation of ETCO_2_ on arrival and lactate concentration

**Fig. 3:**
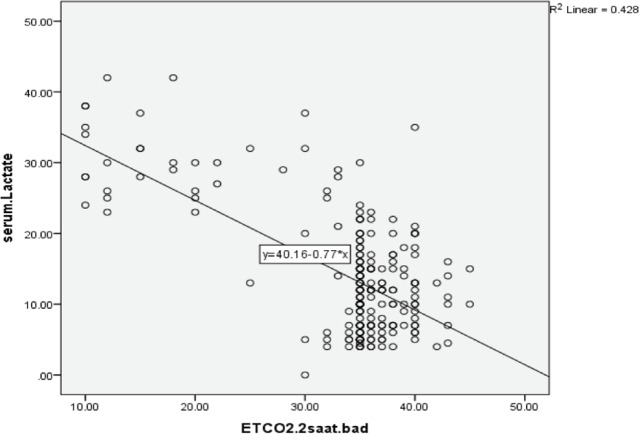
Correlation of ETCO_2_ 2 hours after arrival and lactate concentration

Among 1490 multiple trauma patients admitted to the emergency department, 1240 patients were excluded from the study and 250 patients (male 73.6 % and female 26.4 %) were enrolled in the study (Figure 1). Hospital mortality was reported to be 37 patients (14.8 %). The mean age was 34.18±14.77 (Table 1). In the Pearson correlation, there was a significant inverse relationship between serum lactate and ETCO_2_ immediately (*p*<0.0001, r=-0.65). That is, with increasing serum lactate, ETCO_2_ decreased and as it decreased, the ETCO_2_ increased (Figures 2 and 3).

**Fig. 4:**
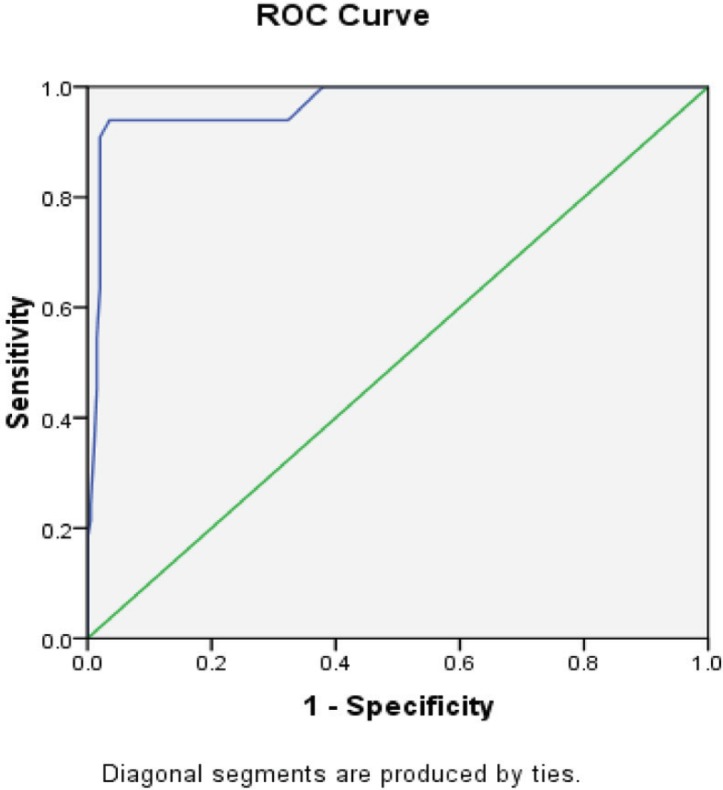
Receiver operating characteristic curve for lactate predicting hospital mortality

**Fig. 5:**
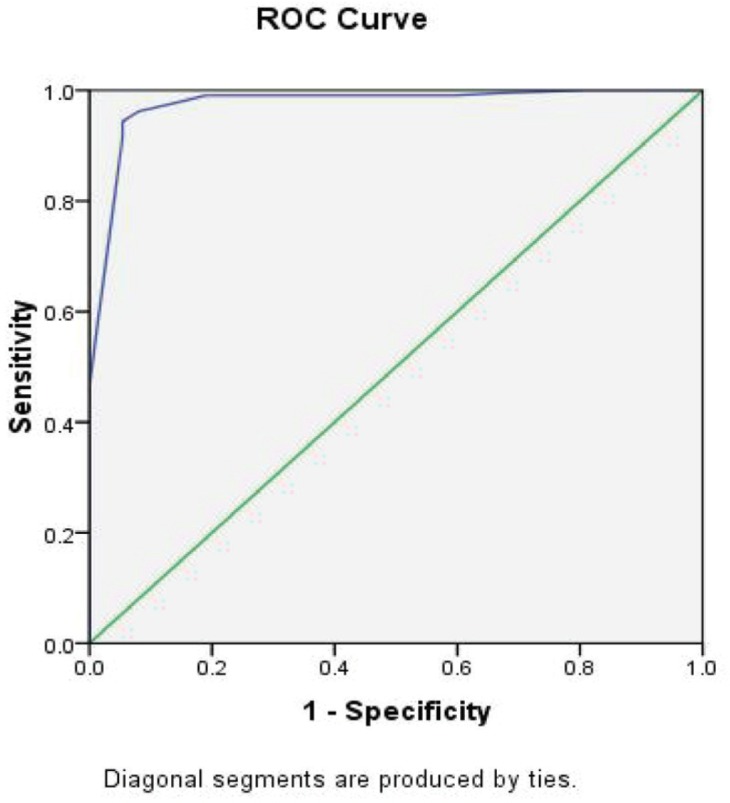
Receiver operating characteristic curve for ETCO_2_ predicting hospital mortality

Then, univariate regression analysis was done. All variables except age and gender (*p*=0.55, *p*=0.36, respectively) had a significant correlation with hospital mortality (Table 2). In the multivariate analysis with the forward conditional method, three variables including heart rate (HR), serum lactate, and ETCO_2_ post-intubation remained in the final model (*p*=0.007, *p*=0.009, *p*=0.023, respectively) had a significant relationship with hospital mortality. For each unit decrease in ETCO_2_ and for each unit increase in heart rate and lactate, hospital mortality increased by 13%, 3%, and 26%, respectively (Table 3). For variables that had a significant statistical correlation with the hospital mortality, a Receiver Operating Characteristic (ROC) curve was drawn. The Area Under Curve (AUC) for three variables including HR, lactate, and ETCO_2_ post-intubation were equal to 0.93, 0.96, 0.97, respectively (Table 4, Figures 4 and 5). 

## Discussion

While there is an inverse relationship between ETCO_2_ and serum lactate in patients with suspected sepsis [14, 15], this correlation is unclear in intubated multiple trauma patients for determining their prognosis. However, our study showed that there was a reverse relationship between ETCO2 and serum lactate in these patients. ETCO_2_ post-intubation and serum lactate both have a predictive role in the hospital mortality in intubated multiple trauma patients. In the emergency department, the differentiation of patients with minor trauma from the major is very difficult and the traditional vital signs are not very helpful for this differentiation [17]. 

One way to evaluate these patients is to examine their physiological status by measuring ETCO_2_. Capnography is easily accessible in emergency departments and is very easy to perform. In intubated patients, capnography is performed using sidestream method by connecting the interface between the tracheal tube and the mechanical ventilation device [18]. ETCO_2_ represents the body's perfusion so that its reduction is proportional to hypoperfusion [19]. In addition, it can also give a physician an important clue about patients’ body acidity [20]. 

The decrease in ETCO_2_ of patients suggests an ominous condition and can predict hospital mortality in patients [20, 21]. Therefore, ETCO_2_ can be helpful in determining the survival rate of patients. This prognostic value has been investigated in some circumstances, such as the confirmation of the presence of an appropriate airway, the confirmation of the return of spontaneous circulation in Cardiopulmonary Resuscitation (CPR), hemodynamic instability, shock and sepsis, and evaluation during surgery [8, 21-23]. 

According to our experience, no study has ever evaluated the relationship between ETCO_2_ and serum lactate in intubated multiple trauma patients in order to determine the prognosis of these patients. In this study, we observed that evaluating ETCO_2_ in immediately post-intubation can have a prognostic role in predicting hospital mortality. The evaluation of serum lactate level is one of the laboratory tests that help determining the prognosis of critically ill patients. It increases under the condition of anaerobic metabolism [14, 15]. This test also increases in trauma patients, which has a direct relationship with hospital mortality [24-27]. Our study, which was conducted on intubated multiple trauma patients, also confirmed this relationship. Carbon dioxide is the product of aerobic metabolism and lactate is the product of anaerobic metabolism. By increasing serum lactate level, the patient experiences lactic acidosis. Furthermore, patients with rapid and deep breathing attempt to compensate for acidosis which results in develops respiratory alkalosis. Therefore, in critically ill patients with a severe reduction in the aerobic metabolism and increase in anaerobic metabolism, ETCO_2_ decreases and lactate levels increase and it is expected that there is a significant reverse relationship between lactate and ETCO_2 _[22,28]. This relationship is helpful in determining the prognosis of these patients, which our study also confirmed this result in intubated multiple trauma patients.

There were several limitations in this study. One of the limitations was the fact that this study was a unicenter study. Non-intubated trauma patients who were less than 18 years old and patients with ISS<16, and pregnant patients were excluded from the study. Those patients who were referred from other hospitals and were late upon their arrivals, and had a chronic pulmonary illness or had drug poisoning were excluded from the study. It was not possible to measure serum lactate at all times. Patients used a different drug prescription in the RSI protocols were excluded from the study.

In conclusion, physiological and metabolic investigations can be helpful in determining the prognosis of critically ill patients. There is a reverse relationship between ETCO_2_ and serum lactate in intubated multiple trauma patients. Both ETCO_2_ and serum lactate have a predictive role in determining the hospital mortality in these patients.

**Table 1:** Baseline characteristics of the patients

Variables	N (%)
Age (Years), mean ± SD	34.18±14.77
Gender	
Male	184 (73.6)
Female	66 (26.4)
HRa (Beats/min), mean ± SD	96.68 ± 23.02
SBPb(mmHg), mean ± SD	115.81 ± 18.87
Lactate (meq/Lit), mean ± SD	13.99 ± 8.66
ETCO2c, mean ± SD	33.71 ± 7.32
Hospital mortality	37 (14.8)

^a^HR: Heart rate; ^b^SBP: Systolic blood pressure; ^c^ETCO_2_: End tidal CO_2_

**Table 2:** Associations with hospital mortality using univariate regression analysis

Variables	**Mortality**			
	**Yes**	**No**	**OR (95% CI)**	**P value**
Age (Years)	33.86±19.06	34.23±13.94	0.94 (0.79-1.10)	0.55
HRa (Beats/min)	132.16±17.64	90.48±17.62	1.12 (0.98-1.28)	<0.0001
SBP_b_ (mmHg)	91.02±21.09	120.06±14.80	1.04 (0.95-1.14)	<0.0001
Base Excess (meq/Lit)	-9.69±4.44	-0.89±7.34	1.00 (0.69-1.48)	<0.0001
Hemoglobin (g/dL)	10.53±1.61	13.96±9.07	1.01 (0.69-1.48)	0.021
INR^c^	1.80±1.49	1.13±0.38	0.54 (0.15-0.00)	<0.0001
Blood sugar (mg/dL)	164.60±57.33	113.14±36.33	1.00 (0.91-1.09)	<0.0001
Lactate (mmol/Lit)	29.30±6.60	11.43±5.90	1.34 (0.87-2.05)	<0.0001
ETCO2^d^	19.59±8.09	36.16±3.32	0.57 (0.26-1.26)	<0.0001
RTS_e_	4.54±1.52	7.09±0.78	0.17 (0.01-1.75)	<0.0001
SS_f_	36.44±5.77	25.38±5.24	1.10 (0.77-1.55)	<0.0001

^a^HR: Heart rate; ^b^SBP: Systolic Blood Pressure; ^c^INR: *International Normalized Ratio;* ^d^ETCO2: End Tidal CO2; ^e^RTS: Revised *Trauma* Score; ^f^ISS: Injury Severity Score.

**Table 3:** Associations with hospital mortality using multivariate regression analysis.

Variable	OR^a^ (95% CI)	P value
Heart rate	1.03 (1.01-1.06)	0.007
Lactate	1.26 (1.06-1.51)	0.009
ETCO_2_^b^	0.87 (0.78-0.98)	0.023

^a^OR: Odss Ration; ^b^ETCO2: End-Tidal CO2

**Table 4:** AUC, Sensitivity, and specificity of variables from the final model

Variable	AUC^a^	Sensitivity (%)	Specificity (%)
HR^b^	0.93	97	81
Lactate	0.96	93	97
ETCO_2_^c^	0.97	85	97

^a^AUC: Area Under Curve; ^b^HR: Heart Rate; cETCO2: End-Tidal CO2

## Conflict of Interest:

None declared.
